# Gonadotropin-releasing hormone agonist for the preservation of ovarian function in survivors of haematopoietic stem cell transplantation for haematological diseases

**DOI:** 10.1186/s12905-022-02039-8

**Published:** 2022-11-07

**Authors:** Zhenhong Wang, Jian An, Chaohua Wang

**Affiliations:** 1 Department of Gynecology, Fujian Maternity and Child Health Hospital College of Clinical Medicine for Obstetrics & Gynecology and Pediatrics, Fujian Medical University, Fuzhou, Fujian 350001 P.R. China; 2 Department of Gynecology, Women and Children’s Hospital, School of Medicine, Xiamen University, Xiamen, 361000 P.R. China; 3 Department of Gynecology and Obstetrics, Peking University People’s Hospital, Beijing, 100044 P.R. China

**Keywords:** Chemotherapy, Premature ovarian failure, Perimenopausal symptoms, Myeloablative regimen, Leukemia

## Abstract

**Objective:**

Administration of GnRH agonist (GnRHa) prior to chemotherapy may decreases the risk of gonadal dysfunction in patients with tumors. However, relevant data in haematopoietic stem cell transplantation (HSCT) recipients has not yet been established. Hence, the present study was designed to evaluate the clinical efficacy of GnRHa cotreatment prior to myeloablative regimens on ovarian protection in female survivors of HSCT for haematological diseases.

**Patients and methods:**

Eligible patients were divided into a GnRHa group and a control group. Medical records regarding age at HSCT; diagnosis/indication for HSCT; pre- and posttransplantation serum sex hormone levels; menstruation and perimenopausal symptoms after HSCT were collected and compared. The primary and secondary outcome was the incidence of premature ovarian insufficiency (POI) symptoms associated with hypoestrogenism.

**Results:**

A total of 330 patients were enrolled in the study: 19 patients were lost to follow-up, and clinical information was obtained in 311 patients. There was no significant difference in the primary outcome of follow-up between the two groups (78.50% [84 of 107] for the GnRHa group versus 83.33% [170 of 204] for the control group). The adjusted relative risks (RR) and 95% confidence interval (CI) were 1.19 and 0.73–1.93 (*P* = 0.487). Among patients who received cotreatment with GnRHa, 62.62% (67 of 107) complained of perimenopausal symptoms, which was significantly lower than the 74.51% (152 of 204) in the control group (adjusted RR: 1.46, 95% CI: 1.04–2.06, *P* = 0.031).

**Conclusion:**

GnRHa cotreatment may not decrease the POI rate in HSCT survivors. However, it may reduce perimenopausal symptoms in this population, suggesting a potential benefit of GnRHa in clinical practice and warrant further researches.

## Introduction

With advances in technology and the increasing numbers of long-term cancer survivors after haematopoietic stem cell transplantation (HSCT), late effects have become the primary concern instead of survival [1–3]. Many haematologic disease survivors of reproductive age face infertility or premature ovarian insufficiency (POI) resulting from exposure to gonadotoxic chemo- and radiotherapy pior to HSCT [4, 5]. Thus, more insight has been gained into protection of the ovary to preserve fertility during cancer-related therapy. For female survivors, several options, including embryo cryopreservation, oocyte cryopreservation, ovarian cryopreservation and transposition, and administration of GnRH agonist (GnRHa) have been proposed for preserving fertility [6–8].

Cytotoxic agents cause the apoptosis of follicles, decreasing the levels of estrogens and inhibin and, accordingly, increased the levels of FSH, which stimulates the recruitment of primordial follicles (the so called “burn-out effect of chemotherapy”) [9]. By binding to GnRH receptors, treatment with GnRH-a leads to a decrease in the secretion of follicle-stimulating hormone (FSH), and luteinizing hormone (LH), which causing a suppression state that keeps the follicles in a quiescent state, making them less vulnerable to chemo-induced damage [9, 10]. Another potential protective mechanism is the reduction of utero-ovarian perfusion after GnRHa administration, which would also reduce the exposure of follicles to the gonadotoxic effect [11]. Furthermore, GnRHa correlate with the upregulation of the anti-apoptotic molecule sphingosine-1-phosphate (S1P), which inhibits the ceramide pathway involved in chemo-induced apoptosis of the ovarian cells and improves neo-angiogenesis in primordial ovarian follicles, resulting in a protective effect in the ovaries [12]. Therefore, according to previous reports, GnRHa cotreatment seems to be an attractive option considering its advantages of safety, noninvasiveness, and feasibility [9, 13].

So far, administration of GnRHa before/during routine chemotherapy may decreases the risk of gonadal dysfunction, and fertility preservation of GnRHa has been investigated in patients with different types of tumors [14–16]. However, the possible utility of this pharmacologic application of preserving ovarian function and fertility in HSCT recipients who have received high chemotherapy dosages in myeloablative regimens and total-body irradiation has not yet been established. Hence, the present study was designed to investigated the benefit of GnRHa on ovarian protection in female survivors of HSCT for haematological diseases, by evaluating the incidence of POI as primary outcome and assessing symptoms associated with hypoestrogenism as secondary outcome.

## Materials and methods

### Study population

We carried out this retrospective cohort study among consecutive female patients who had been referred to gynaecologic endocrinology clinic of Peking University People's Hospital before myeloablative chemotherapy between 2011 and 2018. The inclusion criteria were patients who (a) would undergo allogeneic HSCT for haematological diseases and (b) were postmenarchal and aged < 40 years old [17]. Patients who were found to have decreased ovarian function before myeloablative chemotherapy were excluded. All patients provided written informed consent prior to participating in the study.

We collected the following data from the medical records of the included patients: age at HSCT; diagnosis/indication for HSCT; total body irradiation (TBI) exposure; alkylator agent exposure; pre- and posttransplantation serum levels of FSH, LH, and estradiol (E2) on days 2–4 of menses; menstruation recovery status and perimenopausal symptoms after HSCT; and duration of outpatient follow-up.

### Treatment

Every consecutive patient was offered a monthly depot injection of 3.75 mg of GnRHa before HSCT. And patients didn’t receive GnRHa treatment if they rejected, based on voluntariness. The GnRHa administration was timed as early as possible, usually no longer than 7–14 days before starting chemotherapy [18–21]. For patients for whom the haematologists or oncologists recommended the urgent initiation of chemotherapy, the interval was shorter (< 7 days). Patients were divided into a GnRHa group and a control group according to whether they received GnRHa cotreatment before the conditioning regimen given prior to HSCT.

### Outcome and evaluation criteria

The primary outcome was to evaluate the incidence of POI, which was defined as persistent hypergonadotropic amenorrhea (FSH ≥ 25 U/L on at least two occasions) and low E2 levels. We also defined recovery of cyclic ovarian function (COF) as the spontaneous resumption of menses within one year after transplantation. Patients whose FSH levels did not yet meet the diagnostic criteria of POI but who had been given hormone replacement therapy (HRT) were defined as having unknown outcomes.

As secondary objectives, symptoms associated with hypoestrogenism, including hot flashes, mood swings, sleep disturbance, sexual dysfunction, joint/muscle pain, and dysuria/urinary frequency, were recorded once appear and during regular follow-up. And whether the sypmtoms subside or not wasn’t recorded. Patients who had suffered from these symptoms before the interview were excluded from analysis.

### Statistical analysis

Categorical variables are represented by proportions and percentages, and they were compared between groups with the χ^2^ test (Pearson and Fisher’s exact tests). Continuous variables are summed up by their median and interquartile range (IQR), and comparisons of two groups were performed using Mann–Whitney U tests. Log-binomial regression models were conducted to estimate the relative risks (RR) for outcomes. *P* < 0.05 was considered statistically significant. Statistical analysis was performed using International Business Machines (IBM) Corporation's Statistical Package for Social Sciences (SPSS) version 24.0 (IBM Corporation, Armonk, New York, USA).

## Results

### Clinical characteristics of the study population

Between July 2011 and April 2018, 330 eligible patients were enrolled in the study, 19 patients were lost to follow-up, and clinical information could be obtained in 311 patients: 107 in the GnRHa group and 204 in the control group. There were 15 patients in the GnRHa group who required the initiation of myeloablative chemotherapy with a shorter interval. The shortest follow-up was 3 years, and the longest was 5 years. The basal characteristics of the population are described in Table 1. In the GnRHa group, 64.49% (69 of 107) had standard chemotherapy before the myeloablative regimen, which was significantly lower than the 83.33% (170 of 204) in the control group (*P* < 0.001). There was no significant difference in age, TBI exposure, or pretransplantation serum levels of FSH, LH, and estradiol between the two groups.

**Table 1:** Basal clinical characteristics of the study population

	**GnRHa(107)**	**Control(204)**	***P***
**Age(years)**	22.86 ± 6.59	23.49 ± 6.85	0.435
**Diagnosis for HSCT**			
Lymphoblastic leukemia	32(29.91)	87(42.65)	0.001
Myeloid leukemia	32(29.91)	77(37.75)	
Aplastic anemia	28(26.17)	17(8.33)	
Myelodysplastic syndrome	13(12.15)	19(9.31)	
Others	2(1.87)	4(1.96)	
**Conventional chemotherapy prior to the myeloablative regimen for HSCT**			
Yes	69(64.49)	170(83.33)	< 0.001
No	38(35.51)	34(16.67)	
**TBI exposure**			
Yes	2(1.87)	8(3.92)	0.330
No	105(98.13)	196(96.08)	
**Alkylator agent exposure**	107(100.00)	204(100.00)	/
**Pre-transplantation FSH(mIU/ml)**	8.86 ± 5.27	9.31 ± 4.60	0.338
**Pre-transplantation LH(mIU/ml)**	8.39 ± 6.13	7.45 ± 3.35	0.403
**Pre-transplantation E2(pg/ml)**	82.27 ± 60.43	78.90 ± 37.08	0.614

*HSCT* Hematopoietic stem cell transplantation, *TBI* Total body irradiation, *FSH* Follicle-stimulating hormone, *LH* Luteinizing hormone, *E2* Estradiol

### Primary objectives: incidence of POI and menses recovery

**Fig.1:**
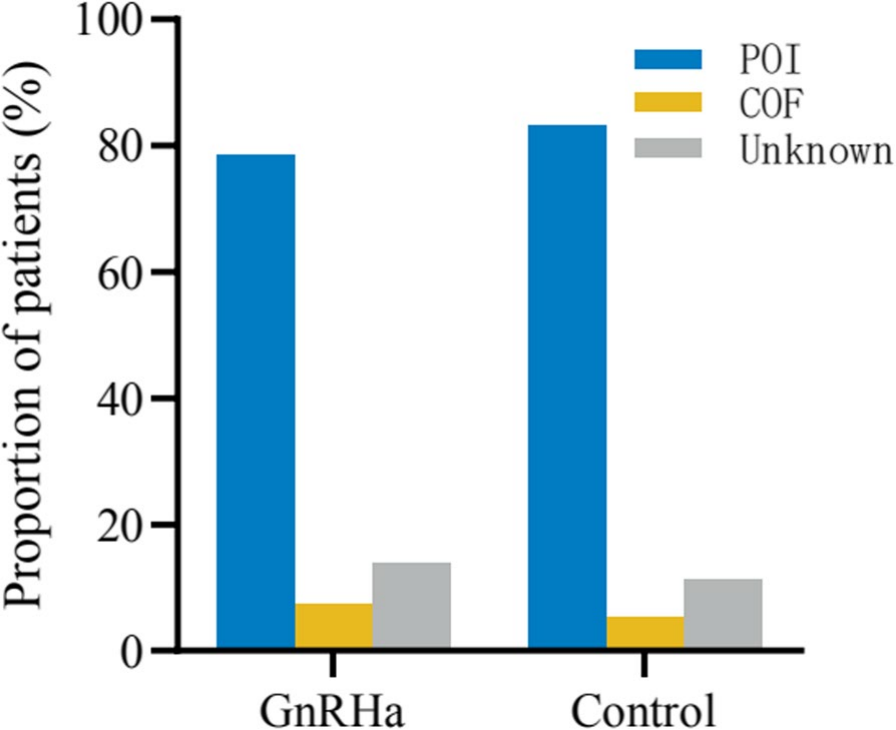
The difference between the GnRH-a treated or untreated patients regarding POI rate in survivors did not reach statistical significance for the study population. POI, premature ovarian insufficiency; COF, cyclic ovarian function

Overall, 81.67% (254 of 311) of the patients had POI; 6.11% (19 of 311) experienced the resumption of menses; and 12.22% (38 of 311) received HRT but did not have spontaneous resumption of menses and yet met the diagnostic criteria of POI. In the GnRHa group, 78.50% (84 of 107) had POI versus 83.33% (170 of 204) in the control group. In contrast, 7.48% (8 of 107) of patients spontaneously resumed menses in the GnRHa group versus 4.39% (11 of 204) in the control group. In the GnRHa group, 14.02% (15 of 107) of patients whose ovarian function was unknown received HRT within one year after HSCT versus 11.27% (23 of 204) in the control group. There was no significant difference in the primary outcome of follow-up between the two groups (*P* = 0.568) (Fig. 1).

**Fig. 2:**
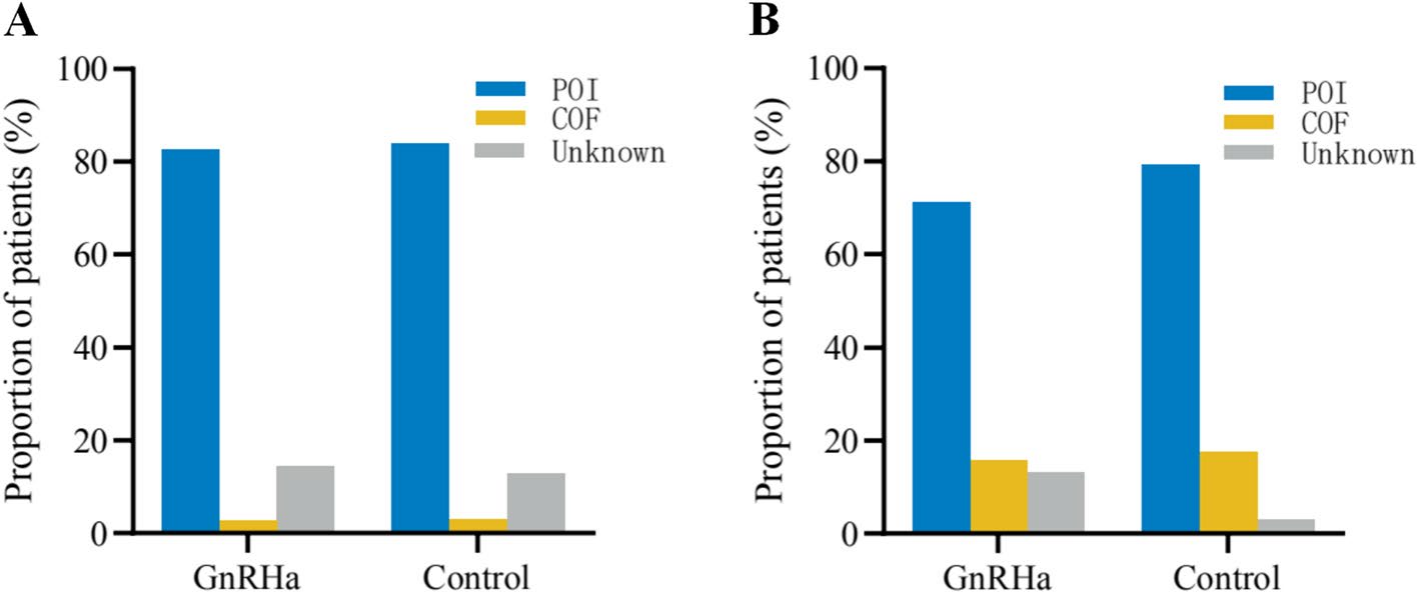
The difference between the GnRH-a treated or untreated patients regarding POI rate in survivors did not reach statistical significance after subgroup analysis based on whether received conventional chemotherapy prior to myeloablative regimen for HSCT (**A**) or not (**B**). POI, premature ovarian insufficiency; COF, cyclic ovarian function

Considering that there were fewer patients who had undergone the conventional chemotherapy prior to the myeloablative regimen in the GnRHa group than in the control group, we performed a subgroup analysis to minimize bias. The results showed that 239 patients received chemotherapy prior to the myeloablative regimen for HSCT. In the GnRHa group, 82.61% (57/69) had POI versus 84.12% (143/170) in the control group, 2.90% (2/69) resumed menses versus 2.94% (5/170) in the control group, and 14.49% (10/69) had an unknown outcome versus 12.94% (22/170) in the control group. The difference between the two groups was not statistically significant (*P* = 0.946) (see Fig. 2a). Among the 72 patients who did not receive chemotherapy prior to myeloablative chemotherapy and HSCT, 71.05% (27/38) had POI versus 79.41% (27/34) in the control group, 15.79% (6/38) resumed menses versus 17.65% (6/34) in the control group, and 13.16% (5/38) had an unknown outcome 2.94% (1/34) in the control group. The difference between the two groups was not statistically significant (*P* = 0.365) (see Fig. 2b).

Log-binomial regression were subsequently applied to determine RR of POI and examine potential influencing factors on POI (Table 2). Compared to women without GnRHa treatment, the unadjusted RR of POI was 1.29(95% CI: 0.80–2.07, *P* = 0.293). There was no statistical significance even after adjusting for factors including conventional chemotherapy prior to the myeloablative regimen for HSCT and TBI exposure. (adjusted RR: 1.19, 95% CI: 0.73–1.93, *P* = 0.487).

**Table 2:** Number of POI after HSCT and RR at 95% CI (patients with GnRHa treatment or without it)

	**GnRHa**	**Control**	**Total**	**RR(95%CI)**^**a**^	***P***
**POI**	84(78.50)	170(83.33)	254(81.67)	1.29(0.80–2.07)	0.293
**Not POI**	23(21.50)	34(16.67)	57(18.33)		
**Total**	107	204	311		

^a^There was no statistical significance even after adjusting for factors. (RR: 1.20, 95% CI: 0.74–1.95, *P* = 0.465), adjusted for conventional chemotherapy prior to the myeloablative regimen for HSCT; (RR: 1.27 95% CI: 0.79–2.05, *P* = 0.322), adjusted for TBI exposure; (RR: 1.19, 95% CI: 0.73–1.93, *P* = 0.487), adjust for above two factors simultaneously

**Fig. 3:**
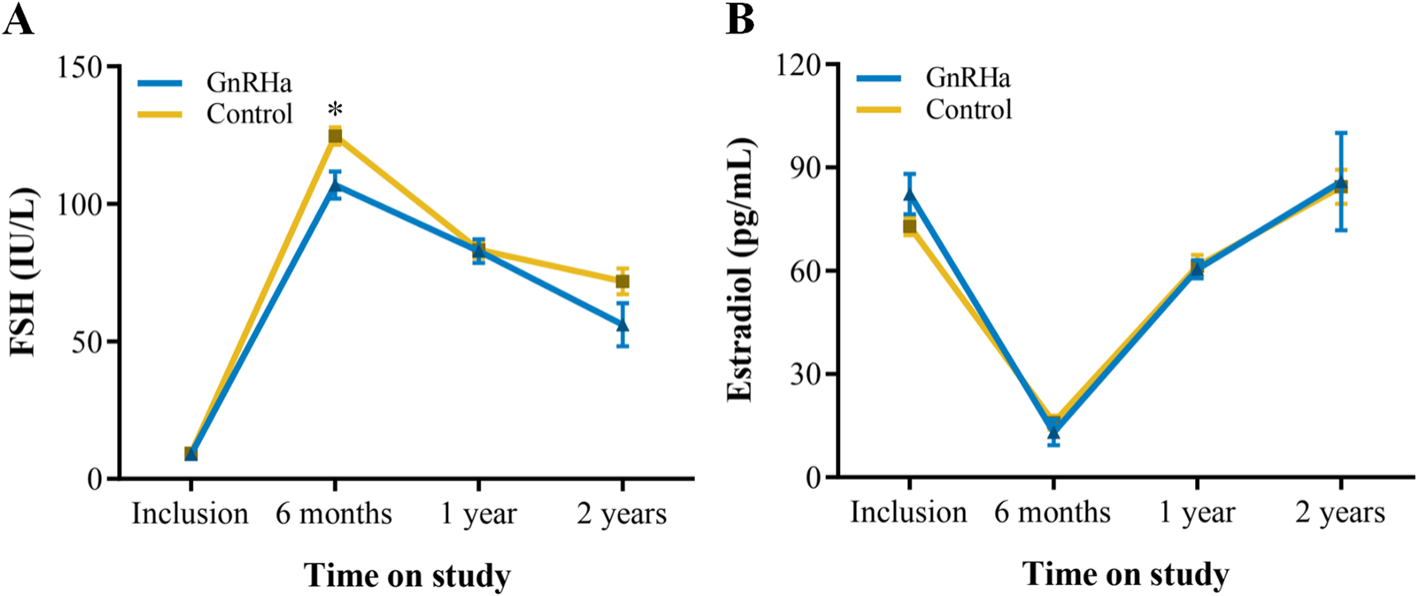
Ovarian function follow-up. Data are presented as mean ± SEM. (**A**) FSH and (**B**) estradiol values at the following time points: at inclusion; at 6 months, 1 and 2 years of follow-up. *P < 0.05. FSH, follicle-stimulating hormone; GnRHa, gonadotropin-releasing hormone agonist

As expected, the mean FSH and E2 levels were significantly lower in the GnRHa group than in the control group after HSCT, but this difference was no longer observed during the long-term follow-up (Fig. 3).

### Secondary objectives: perimenopausal symptoms

Perimenopausal symptoms associated with hypoestrogenism were reported in 70.42% (219 of 311) of patients. Among patients who received cotreatment with GnRHa, 62.62% (67 of 107) complained of perimenopausal symptoms, which was significantly lower than the 74.51% (152 of 204) in the control group (RR: 1.47, 95% CI: 1.05–2.06, *P* = 0.027). The difference remained statistically significant after adjusting for factors including conventional chemotherapy prior to the myeloablative regimen for HSCT (RR: 1.488, 95% CI: 1.055–2.098, *P* = 0.024) and TBI exposure (RR: 1.44, 95% CI: 1.02–2.02, *P* = 0.036; adjust for two factors simultaneously, RR: 1.46, 95% CI: 1.04–2.06, *P* = 0.031).

Of the 6 menopausal symptoms, the order of reported symptoms was mood swings (71.64%, 48 of 67), hot flashes (58.21%, 39 of 67), sleep disturbance (29.85%, 20 of 67), sexual dysfunction (26.87%, 18 of 67), joint/muscle pain (2.99%, 2 of 67), and dysuria/urinary frequency (1.49%, 1 of 67) in the GnRHa group and hot flashes (61.18%, 93 of 152), mood swings (60.53%, 92 of 152), sleep disturbance (39.47%, 60 of 152), sexual dysfunction (35.53%, 54 of 152), joint/muscle pain (12.50%, 19 of 152), and dysuria/urinary frequency (2.63%, 4 of 152) in the control group (Table 3).

**Table 3:** Number of second perimenopausal symptoms after HSCT and RR at 95% CI (patients with GnRHa treatment or without it)

	**GnRHa(107)**	**Control(204)**	**RR(95%CI)**^**a**^	***P***
**Perimenopausal symptoms**				
** Yes**	67(62.62)	152(74.51)	1.47(1.05–2.06)	0.027
Hot flashes	39	93		
Mood swings	48	92		
Sleep disturbance	20	60		
Sexual dysfunction	18	54		
Joint/muscle pain	2	19		
Dysuria/urinary frequency	1	4		
**No**	40(37.38)	52(25.49)		

^a^The difference remained statistically significant after adjusting for factors. (RR: 1.488, 95% CI: 1.055–2.098, *P* = 0.024), adjusted for conventional chemotherapy prior to the myeloablative regimen for HSCT; (RR: 1.44, 95% CI: 1.02–2.02, *P* = 0.036), adjusted for TBI exposure; (RR: 1.46, 95% CI: 1.04–2.06, *P* = 0.031), adjust for above two factors simultaneously

## Discussion

At present, GnRHa treatment is not supported as a proven method for fertility preservation and is considered experimental, with reference to international guidelines [22–24]. Our study did not demonstrate that GnRHa could significantly reduce the incidence of POI in HSCT survivors. To date, only a few studies have addressed the issue of GnRHa efficacy in terms of ovarian protection in HSCT patients to date [25–27]. The results from previous studies have generated a debate concerning the efficacy of GnRHa in fertility preservation after HSCT. Relevant to this highly controversial issue, Blumenfeld et al [25] compared the incidence of POI after HSCT in young women who received GnRHa cotreatment versus those who did not. The study included 83 patients, and the results showed that the administration of GnRHa increased the rate of cyclic ovarian function in the HSCT survivors from 11 to 38% and decreased the rate of POI from 89 to 62% (*P* = 0.006). They concluded that GnRHa cotreatment may be beneficial in women undergoing HSCT, especially for lymphoma, providing convincing evidence in support of the efficacy of this preventive strategy. In another prospective study reported by Cheng and coworkers [26], forty four evaluable patients were treated with two doses of 22.5 mg leuprolide before HSCT. After follow-up for a median of 355 days, the researchers found that normal ovarian function was restored in 16% (7/44) of women: 18% (6/33) in the myeloablative group and 9% (1/11) in the nonmyeloablative group (*P* = 0.66). The authors concluded that GnRHa did not preserve ovarian function in patients who underwent HSCT using either myeloablative or nonmyeloablative regimens. There may be several reasons for the different results generated by the aforementioned similar works. First, the diagnostic criteria for POI were different in their studies. The study by Blumenfeld et al. defined POI as hypergonadotropin amenorrhea (menopause > 6 months, FSH > 40 U/L), while ovarian failure after transplantation was defined by Cheng and coworkers as the continuation of menstruation cessation for > 3 months in a patient who was premenopausal at the beginning of the study with the following hormonal parameters: (a) serum FSH level > 20 IU/L, (b) serum LH level > 20 IU/L, and (c) serum estradiol level > 30 pmol/L. The diagnostic threshold for FSH is lower and the follow-up time is shorter in Cheng’s research, which indicates insufficient recovery time for ovarian function, leading to a higher incidence of POI. Second, the dosage of GnRHa was different in two studies. Cheng and coworkers administered a higher dose of triptorelin/leuprolide, which led to a 15% (9/59) dropout because of the high rate of intolerable side effects. Third, patients with haematological malignancies in the study by Blumenfeld et al. had started GnRHa cotreatment before first exposure to chemotherapeutic agents. However, GnRH-a was administered before myeloablative chemotherapy preceding HSCT, and almost all patients received at least one prior chemotherapy regimen. This means that the patients were not protected by GnRHa during chemotherapy prior to the myeloablative regimen and were directly exposed to gonadotoxic chemotherapy drugs, which would have inevitably lead to a higher incidence of POI. Additionally, other studies have discussed the clinical efficacy of GnRHa in survivors of HSCT for haematological diseases [27–29]. Nevertheless, their reference value is limited due to the small objects.

Despite the fact that the calculated RR of POI in our study was not statistically significant. The following aspects still deserve to be discussed. The incidence of POI in both the GnRHa group and the control group was lower than the incidence reported in the literature. The main reason is that patients who did not meet the primary end point yet resumed menses after 1 year of follow-up were given HRT therapy thereafter. Therefore, it is impossible to determine whether spontaneous menstruation was restored in patients with unknown outcomes, and some patients whose hormone levels could not meet the diagnostic criteria for POI in our study may have recovered cystic ovarian function. In terms of the timing of GnRHa administration, all patients in this study were given GnRHa before the initiation of myeloablative chemotherapy, and some of the patients in the study population received unprotected gonadal chemotherapy before GnRHa was administered. To eliminate potential bias, we performed a stratified analysis, and the results failed to support the efficacy of GnRHa in HSCT survivors with or without chemotherapy prior to myeloablation. Additionally, it should be noted that hypoestrogenism, caused by POI, is resulting in perimenopausal symptoms, which can have a significant impact on quality of life. Therefore, perimenopausal symptoms and signs of estrogen deficiency are also important reference indicators for evaluating the ovarian protection function of GnRHa. Our study found that GnRHa was able to significantly reduce the incidence of perimenopausal symptoms in HSCT survivors. In addition, although GnRHa does not show the benefits of ovarian protection from the perspective of POI in the present study, we found that FSH levels after HSCT were significantly lower in the GnRHa group than in the control group, indicating a potential effect of ovarian preservation. Thus, GnRHa for the preservation of ovarian function in HSCT survivors is promising and worthy of further investigation.

The main strength of our study is the large-scale population. To the best of our knowledge, this is the largest retrospective study reporting GnRHa cotreatment in HSCT survivors. The main weakness of studies evaluating the role of GnRHa in preserving ovarian function is the retrospective design, with all its inherent risks of bias, which can negatively impact the veracity of the results. The lack of data concerning chemotherapy prior to myeloablative regimens, and uncollected important variables such as BMI, serum AMH level and AFCs, were also both other limitations.

## Conclusion

Based on the above, our study did not provide evidence that GnRHa is effective in preventing POI in HSCT survivors. However, it may reduce perimenopausal symptoms in this population, suggesting a potential benefit of GnRHa preservation of ovarian function. We cautiously suggest that GnRHa cotreatment should be considered in clinical practice. These results warrant further examination by researches with prospective design in the future.

## Data Availability

The datasets used and/or analysed during the current study available from the corresponding author on reasonable request.
